# Effects of cognate, non-cognate and synthetic CXCR4 and ACKR3 ligands on human lung endothelial cell barrier function

**DOI:** 10.1371/journal.pone.0187949

**Published:** 2017-11-10

**Authors:** You-Hong Cheng, Jonathan M. Eby, Heather M. LaPorte, Brian F. Volkman, Matthias Majetschak

**Affiliations:** 1 Burn and Shock Trauma Research Institute, Department of Surgery, Loyola University Chicago Stritch School of Medicine, Maywood, IL, United States of America; 2 Department of Biochemistry, Medical College of Wisconsin, Milwaukee, WI, United States of America; 3 Department of Molecular Pharmacology and Therapeutics, Loyola University Chicago Stritch School of Medicine, Maywood, IL, United States of America; Medical College of Georgia, Augusta, UNITED STATES

## Abstract

Recent evidence suggests that chemokine CXCL12, the cognate agonist of chemokine receptors CXCR4 and ACKR3, reduces thrombin-mediated impairment of endothelial barrier function. A detailed characterization of the effects of CXCL12 on thrombin-mediated human lung endothelial hyperpermeability is lacking and structure-function correlations are not available. Furthermore, effects of other CXCR4/ACKR3 ligands on lung endothelial barrier function are unknown. Thus, we tested the effects of a panel of CXCR4/ACKR3 ligands (CXCL12, CXCL11, ubiquitin, AMD3100, TC14012) and compared the CXCR4/ACKR3 activities of CXCL12 variants (CXCL12α/β, CXCL12(3–68), CXCL12_1_, CXCL12_2_, CXCL12-S-S4V, CXCL12-R47E, CXCL12-K27A/R41A/R47A) with their effects on human lung endothelial barrier function in permeability assays. CXCL12α enhanced human primary pulmonary artery endothelial cell (hPPAEC) barrier function, whereas CXCL11, ubiquitin, AMD3100 and TC14012 were ineffective. Pre-treatment of hPPAEC with CXCL12α and ubiquitin reduced thrombin-mediated hyperpermeability. CXCL12α-treatment of hPPAEC after thrombin exposure reduced barrier function impairment by 70% (EC_50_ 0.05–0.5nM), which could be antagonized with AMD3100; ubiquitin (0.03–3μM) was ineffective. In a human lung microvascular endothelial cell line (HULEC5a), CXCL12α and ubiquitin post-treatment attenuated thrombin-induced hyperpermeability to a similar degree. CXCL12(3–68) was inefficient to activate CXCR4 in Presto-Tango β-arrestin2 recruitment assays; CXCL12-S-S4V, CXCL12-R47E and CXCL12-K27A/R41A/R47A showed significantly reduced potencies to activate CXCR4. While the potencies of all proteins in ACKR3 Presto-Tango assays were comparable, the efficacy of CXCL12(3–68) to activate ACKR3 was significantly reduced. The potencies to attenuate thrombin-mediated hPPAEC barrier function impairment were: CXCL12α/β, CXCL12_1_, CXCL12-K27A/R41A/R47A > CXCL12-S-S4V, CXCL12-R47E > CXCL12_2_ > CXCL12(3–68). Our findings indicate that CXCR4 activation attenuates thrombin-induced lung endothelial barrier function impairment and suggest that protective effects of CXCL12 are dictated by its CXCR4 agonist activity and interactions of distinct protein moieties with heparan sulfate on the endothelial surface. These data may facilitate development of compounds with improved pharmacological properties to attenuate thrombin-induced vascular leakage in the pulmonary circulation.

## Introduction

Acute respiratory distress syndrome (ARDS) remains a major contributor to morbidity and mortality in critically ill patients [1–4]. It is generally accepted that mild ARDS and its progression into moderate and severe ARDS is caused by local and systemic coagulation and inflammation, which leads to impaired pulmonary endothelial barrier function, third spacing of fluids into the lung and formation of lung edema, the hallmark of ARDS [5, 6]. Thrombin plays an important role in the pathogenesis of ARDS; in addition to functions of thrombin in the clotting cascade, thrombin fulfills diverse roles in inflammation and is well known to impair endothelial barrier function through activation of the G protein-coupled receptors (GPCR) protease-activated receptors (PARs) [7–9]. All four members of the PAR family (PAR1-4) can be activated by thrombin [8–10]. A large body of evidence suggests that PAR-1 is the major mediator of thrombin signaling in vascular endothelial cells [8, 11–14]. PAR-1 is activated when thrombin cleaves its extracellular N-terminal domain between residues Arg-41 and Ser-42, which unmasks a new N-terminus that serves as a tethered ligand [8]. Drugs that limit impairment of the lung endothelial barrier by thrombin, however, are not available, but desirable for their potential to improve outcomes.

Recently, administration of cognate, non-cognate and synthetic chemokine (C-X-C) motif receptor (CXCR) 4 agonists has been shown to attenuate lung injury in various experimental models and CXCL12 (stromal cell-derived factor-1α), the cognate agonist of CXCR4 and atypical chemokine receptor 3 (ACKR3), has been described to attenuate thrombin-induced impairment of endothelial cell barrier function [15–22]. A more detailed pharmacological characterization of these CXCL12-mediated effects, however, is lacking and the effects of other CXCR4/ACKR3 ligands on lung endothelial cell barrier function are ill defined. Moreover, information on the structural requirements of CXCL12 to attenuate thrombin-mediated lung endothelial barrier disruption and the relationship to its CXCR4/ACKR3 agonist activity is not available. Because such data could guide the development of compounds with improved efficacy to reduce thrombin-mediated vascular leakage, we tested the effects of a panel of CXCR4/ACKR3 ligands on the barrier function of human lung vascular endothelial cells. We also compared CXCR4 and ACKR3 activities with effects on thrombin-induced lung endothelial barrier disruption of the wild-type splice variants CXCL12α and CXCL12β, of N-terminal truncated CXCL12 (3–68), a posttranslational modification that occurs in vivo after cleavage of CXCL12 by CD26/dipeptidyl peptidase 4, and of several engineered CXCL12 variants and mutants. These proteins have previously been reported to possess distinct affinities for CXCR4/ACKR3 and altered pharmacological properties [23–31]. Thus, we hypothesized that CXCR4 agonists antagonize thrombin-mediated impairment of lung endothelial cell barrier function and that the engineered CXCL12 variants and mutants show distinct biological activities.

## Materials and methods

### Proteins, peptides and reagents

AMD3100 was purchased from Sigma-Aldrich, CXCL12 and CXCL11 from Protein Foundry, ubiquitin from R&D Systems, TC14012 from Tocris Bioscience and human alpha thrombin from Enzyme Research Laboratories. Recombinant CXCL12 variant proteins were expressed in *E*. *coli*, refolded, purified and verified by NMR and high-resolution mass spectrometry as previously described [32]

### Cells and cell lines

Human primary pulmonary artery endothelial cells (hPPAEC) (ATCC, PCS-100-022) and the human lung microvascular endothelial cell line HULEC-5a (ATCC, CRL-3244) were cultured in vascular cell basal medium (ATCC, PCS-100-030) with endothelial cell growth kit-VEGF (ATCC, PCS-100-041). The HTLA cell line, a HEK293 cell line stably expressing a tTA-dependent luciferase reporter and a β-arrestin2-TEV fusion gene [33], was generously provided by the laboratory of Dr. Bryan Roth and maintained in high glucose Dulbecco’s Modified’s Eagle’s Medium supplemented with 10% (vol/vol) FBS, 1x non-essential amino acids, 100 U/mL penicillin, 100 μg/mL streptomycin, 50 μg/mL hygromycin B, and 2 μg/mL puromycin. All cells were cultured at 37°C, 5% CO_2_ in a humidified atmosphere.

### In vitro vascular permeability assays

Permeability assays were obtained from Millipore (ECM642) and performed as per manufacturer’s instructions. In brief, 96-well collagen-coated permeability assay plates were pre-hydrated for 15 min, 5x10^5^ cells were seeded on each well and grown to a confluent monolayer for 48 hours. Fluorescein isothiocyanate (FITC)-dextran (20 μg/mL) was then added on top of the monolayer and the amount of FITC-dextran that permeated through the monolayer was quantified by measuring fluorescence in a Synergy 2 Multi-mode Microplate Reader (BioTek, Winooski, VT) at various time points over a 255 min time period.

### Proximity ligation assays (PLA)

PLA were performed as described in detail previously [34–36], utilizing mouse anti-ACKR3 (R&D MAB42273) and goat anti-CXCR4 (Abcam Ab1670). The antibodies have been validated for their receptor target previously [35–37]. PLA signals (λ_excitation/emission_ 598/634 nm)) were identified as red spots under a fluorescence microscope.

### Presto-Tango β-arrestin 2 recruitment assay

The PRESTO-Tango (parallel receptorome expression and screening via transcriptional output, with transcriptional activation following arrestin translocation) assay was performed as recently described [33]. The Tango plasmids were a gift from Dr. Bryan Roth (all from Addgene). HTLA cells (2.5x10^5^/well) were seeded in a 6-well plate and transfected with 1.5 μg of the Tango plasmids using Lipofectamine 3000 (ThermoScientific). The following day, transfected HTLA cells (1x10^5^ cells/well) were plated onto Poly-L-Lysine pre-coated 96-well microplates and allowed to attach to the plate surface for at least 4 hours prior to treatment. Proteins used for treatment were prepared in twice the final concentration in culture media, added at a 1:1 vol/vol ratio and incubated overnight at 37°C, 5% CO_2_ in a humidified environment. The following morning, media was removed from cell culture plates and replaced with a 100 μL 1:5 mixture of Bright-Glo (Promega) and 1x HBSS, 20 mM HEPES solution. Plates were then incubated at room temperature before measuring luminescence on a Biotek Synergy II plate reader.

### SDS-polyacrylamide gel electrophoresis (PAGE)

SDS-PAGE was performed utilizing pre-cast mini-PROTEAN TGX gels (Bio-Rad). Lanes were loaded with 1ug of each protein in 25 μL of Laemmli sample buffer with or without 10% 2-mercaptoethanol (SigmaAldich) after boiling for 5 min.

### Data analyses

Data are expressed as mean ± SEM from *n* independent experiments that were performed on different days. Data were analyzed with unpaired Student’s *t* test, one- or two-way analyses of variance with Bonferroni’s multiple comparison post hoc test, as appropriate. Dose–response curves were generated using nonlinear regression analyses. All analyses were performed with the GraphPad-Prism 7 software. A two-tailed *P* < 0.05 was considered significant.

## Results

**Fig 1:**
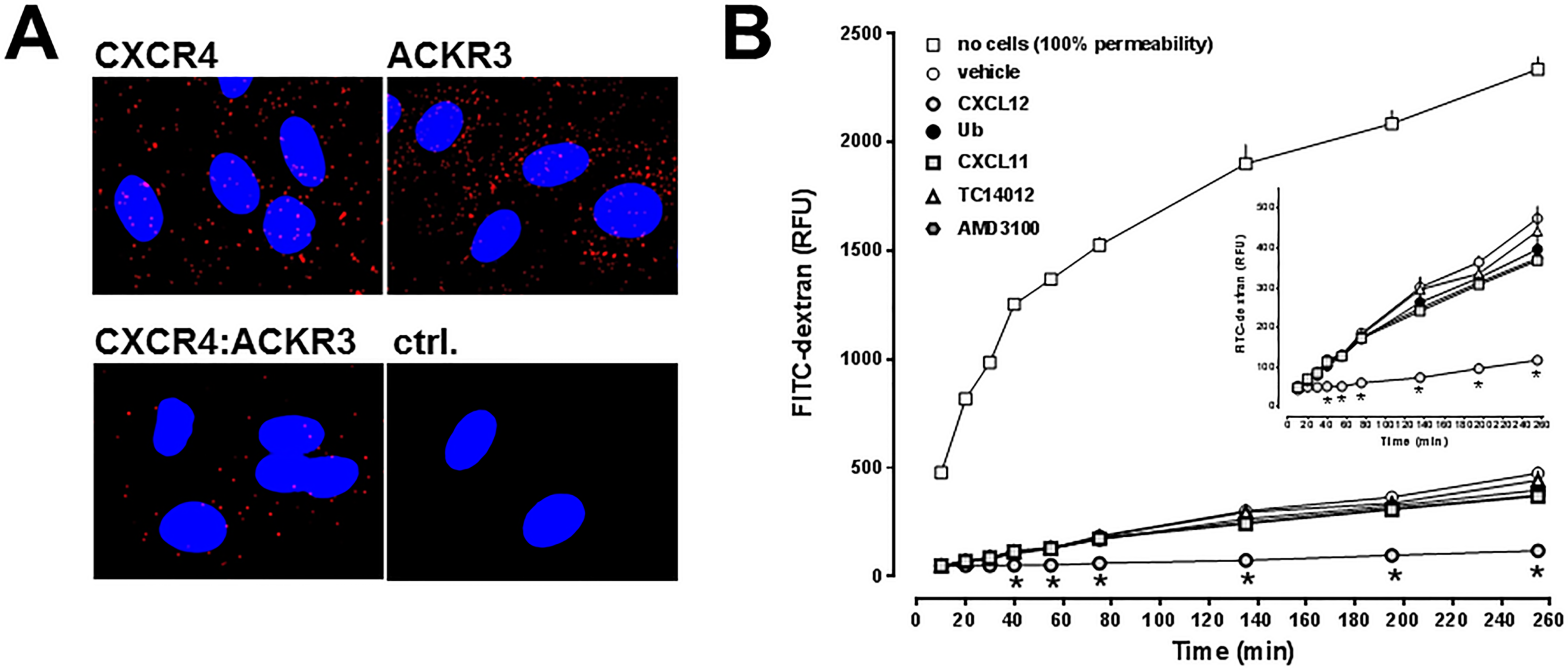
Expression of CXCR4, ACKR3 and CXCR4:ACKR3 heteromers on hPPAEC and effects of CXCR4/ACKR3 ligands on hPPAEC monolayer permeability. (A) Detection of CXCR4, ACKR3 and CXCR4:ACKR3 heteromers on hPPAEC by PLA. Typical PLA images for the detection of individual receptors and CXCR4:ACKR3 heteromers. Images show merged PLA/4′,6-diamidino-2-phenylindole dihydrochloride (DAPI) signals. Ctrl.: Omission of one secondary antibody. (B) hPPAEC were grown to a confluent monolayer on collagen-coated permeable membranes. Cells were then exposed to vehicle or 50 nM of CXCR4/ACKR3 ligands for 10 minutes, as indicated, followed by the addition of FITC-dextran. Endothelial permeability was assessed by measuring the amount of FITC-dextran that permeated through the cell monolayer. N = 3 in quadruplicate. No cells: 100% permeability, open squares. RFU: Relative fluorescence units. *: p<0.05 vs. vehicle (2-way ANOVA/Bonferroni’s multiple comparison post hoc test).

To confirm that CXCR4 and ACKR3 are expressed in hPPAEC and to assess whether both receptors form heteromeric complexes, we performed PLA to detect individual receptors and receptor-receptor interactions at single molecule resolution. As shown in Fig 1A, we observed positive PLA signals for both receptors individually and for CXCR4:ACKR3 heteromeric complexes. We then tested the effects of a panel of CXCR4 and ACKR3 ligands on hPPAEC monolayer permeability in transwell-permeability assays with FITC-dextran (Fig 1B). Ubiquitin, a non-cognate CXCR4 agonist that does not bind to ACKR3, CXCL11, an ACKR3 and CXCR3 agonist, TC14012, a synthetic CXCR4 antagonist and ACKR3 agonist, and AMD3100, a CXCR4 antagonist and allosteric ACKR3 agonist, did not affect hPPAEC permeability [26, 38–41]. In contrast, CXCL12 enhanced hPPAEC barrier function.

**Fig 2:**
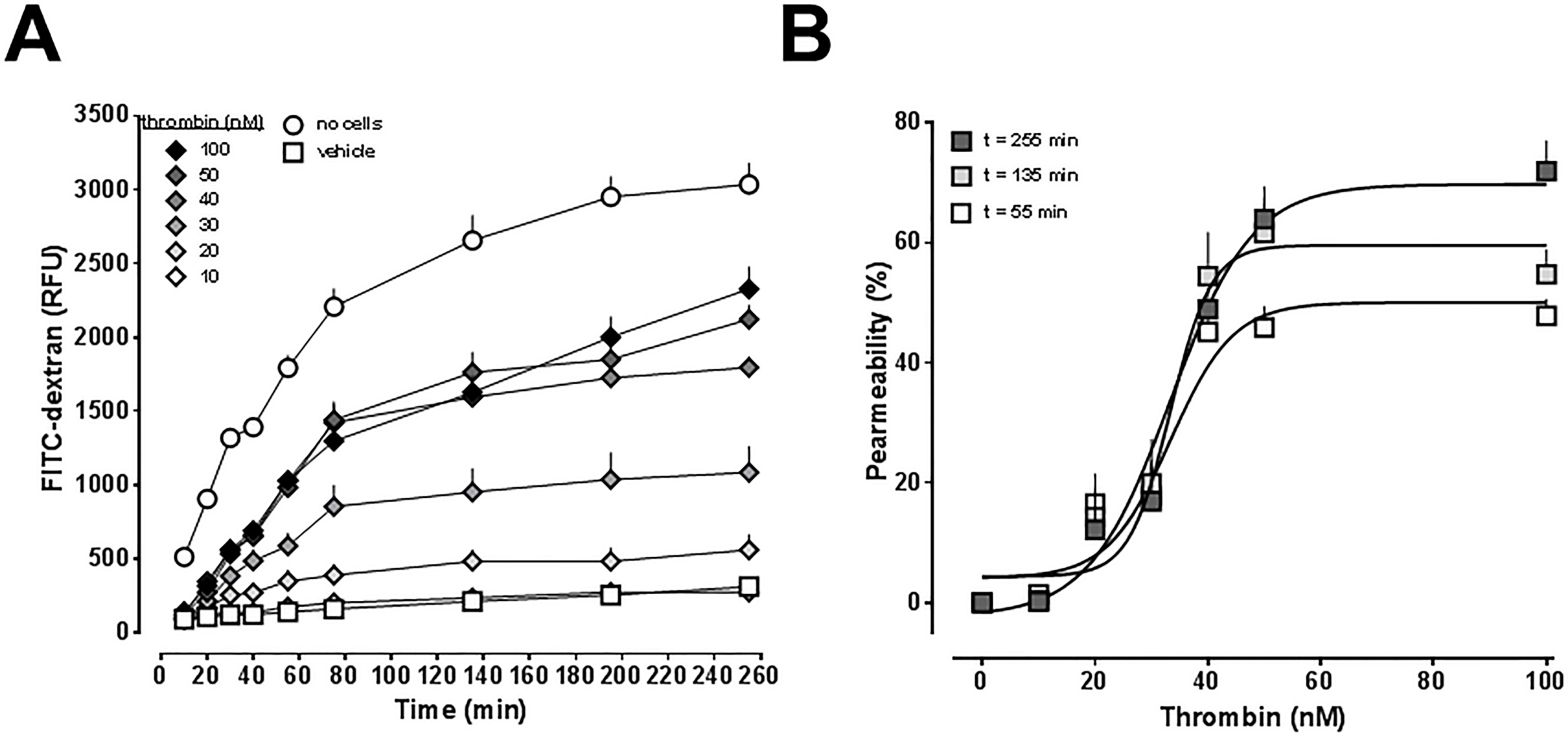
Impairement of hPPAEC monolayer permeability by thrombin. (A) hPPAEC were grown to a confluent monolayer on collagen-coated permeable membranes and then exposed to different concentration of thrombin for 10 min, followed by the addition of FITC-dextran. Endothelial permeability was assessed by measuring the amount of FITC-dextran that permeated through the cell monolayer. No cells: 100% permeability. RFU: Relative fluorescence units. N = 3 in quadruplicate. (B) Dose-response curves for thrombin-induced permeability, data from A. 100% permeability = permeability in the absence of hPPAEC. Open squares: Permeability at t = 55 min. Light grey squares: Permeability at t = 135 min. Dark grey squares: Permeability at t = 255 min. Dose-response curves were generated using nonlinear regression analyses.

To be able to assess the effects of CXCL12 on thrombin-induced impairment of hPPAEC barrier function under optimized conditions, we determined the dose-response characteristics for thrombin in the permeability assay. The effects of thrombin (10–100 nM) on hPPAEC monolayer permeability are shown in Fig 2A. Thrombin dose- and time-dependently induced permeability of the hPPAEC monolayer. The time to reach plateau for the permeability-inducing effects of thrombin increased with increasing thrombin concentrations (20 nM– 55 min; 30 nM– 75 min; 40 nM– 135 min; 50 nM and 100 nM—>255 min). Based on the results from Fig 2A, we analyzed the dose-effect relationship for thrombin-induced impairment of lung vascular endothelial cell barrier function at 55 min, 135 min and 255 min (Fig 2B). The thrombin-mediated effects showed a sigmoidal dose-effect relationship at all time points. The EC_50_ for thrombin-induced impairment of hPPAEC barrier function was 30±2 nM after 55 min, 33±2 nM after 135 min and 36±2 nM after 255 min. The maximal impairment of endothelial barrier function (100% permeability = measured permeability in the absence of hPPAEC) reached 49±3% at 55 min and 59±4% and 72±4% at 135 min and 255 min, respectively.

**Fig 3:**
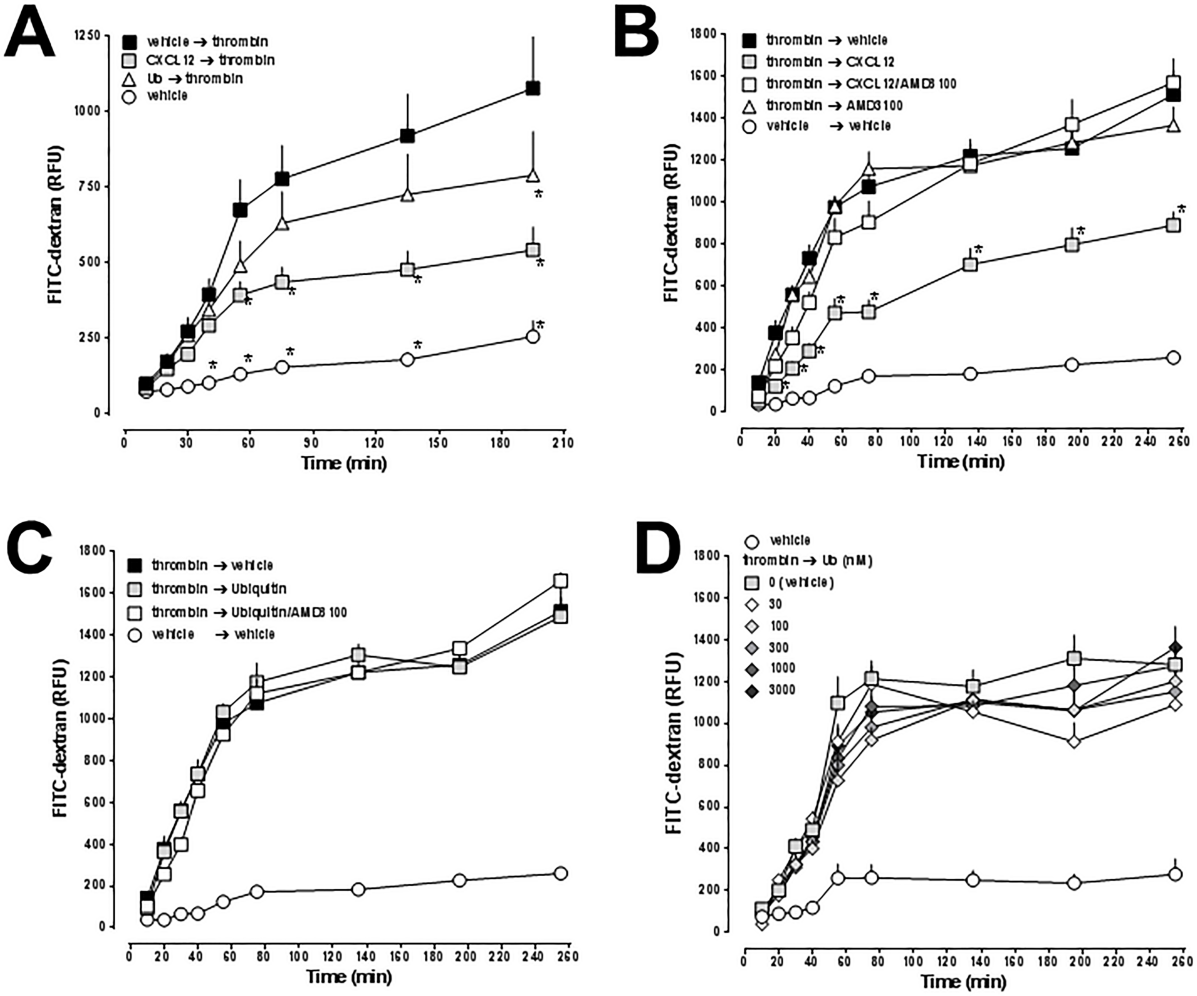
Effects of CXCL12 and ubiquitin on thrombin-induced impairment of hPPAEC monolayer permeability. hPPAEC were grown to a confluent monolayer on collagen-coated permeable membranes. (A) hPPAEC were pre-treated with vehicle, 100 nM of CXCL12 or ubiquitin for 10 minutes, as indicated, and then exposed to thrombin (50 nM), followed by the addition of FITC-dextran. Vehicle: no thrombin. Endothelial permeability was assessed by measuring the amount of FITC-dextran that permeated through the cell monolayer. No cells: 100% permeability (open circles). RFU: Relative fluorescence units. N = 3 in quadruplicate. *: p<0.05 vs. vehicle/thrombin. (B-D) hPPAEC were exposed to 35 nM of thrombin or vehicle. After 10 min, thrombin-exposed cells were treated with vehicle, CXCL12 (50 nM) and/or AMD3100 (10 μM) (B), with vehicle, ubiquitin (50 nM) and/or AMD3100 (10 μM) (C) or with various concentrations of ubiquitin (D) followed by the addition of FITC-dextran. The experimental conditions are indicated. Endothelial permeability was assessed by measuring the amount of FITC-dextran that permeated through the cell monolayer. RFU: Relative fluorescence units. N = 3 in quadruplicate. *: p<0.05 vs. thrombin/vehicle (2-way ANOVA/Bonferroni’s multiple comparison post hoc test).

We then tested whether pre-treatment with CXCL12 and ubiquitin influences hyper-permeability of hPPAEC induced by a sub-maximal dose of thrombin. hPPAEC were treated for 10 min with either 100 nM of CXCL12 or ubiquitin, followed by the addition of of thrombin (Fig 3A). Pre-treatment with both CXCR4 agonists significantly attenuated thrombin-induced hyper-permeability of hPPAEC. CXCL12 was more efficacious than ubiquitin in this assay. The effects of CXCL12 and ubiquitin when added after thrombin treatment of hPPAEC are shown in Fig 3B and 3C. CXCL12 significantly attenuated thrombin-induced permeability of hPPAEC and this effect could be antagonized with the CXCR4 antagonist AMD3100. AMD3100 treatment alone did not affect thrombin-mediated hyper-permeability of hPPAEC (Fig 3B). In contrast to CXCL12, ubiquitin-treatment and ubiquitin plus AMD3100-treatment did not modulate thrombin-induced hyper-permeability when tested in parallel experiments (Fig 3C). To exclude that the dose-effect relationship for ubiquitin is different from the dose-effect relationship for CXCL12, we tested ubiquitin in various concentrations (30 nM– 3 μM), including concentrations above the *K*_D_-value of ubiquitin for CXCR4 binding [39]. Ubiquitin treatment, however, did not attenuate thrombin-induced hyper-permeability of the hPPAEC monolayer at any tested concentration under these experimental conditions (Fig 3D).

**Fig 4:**
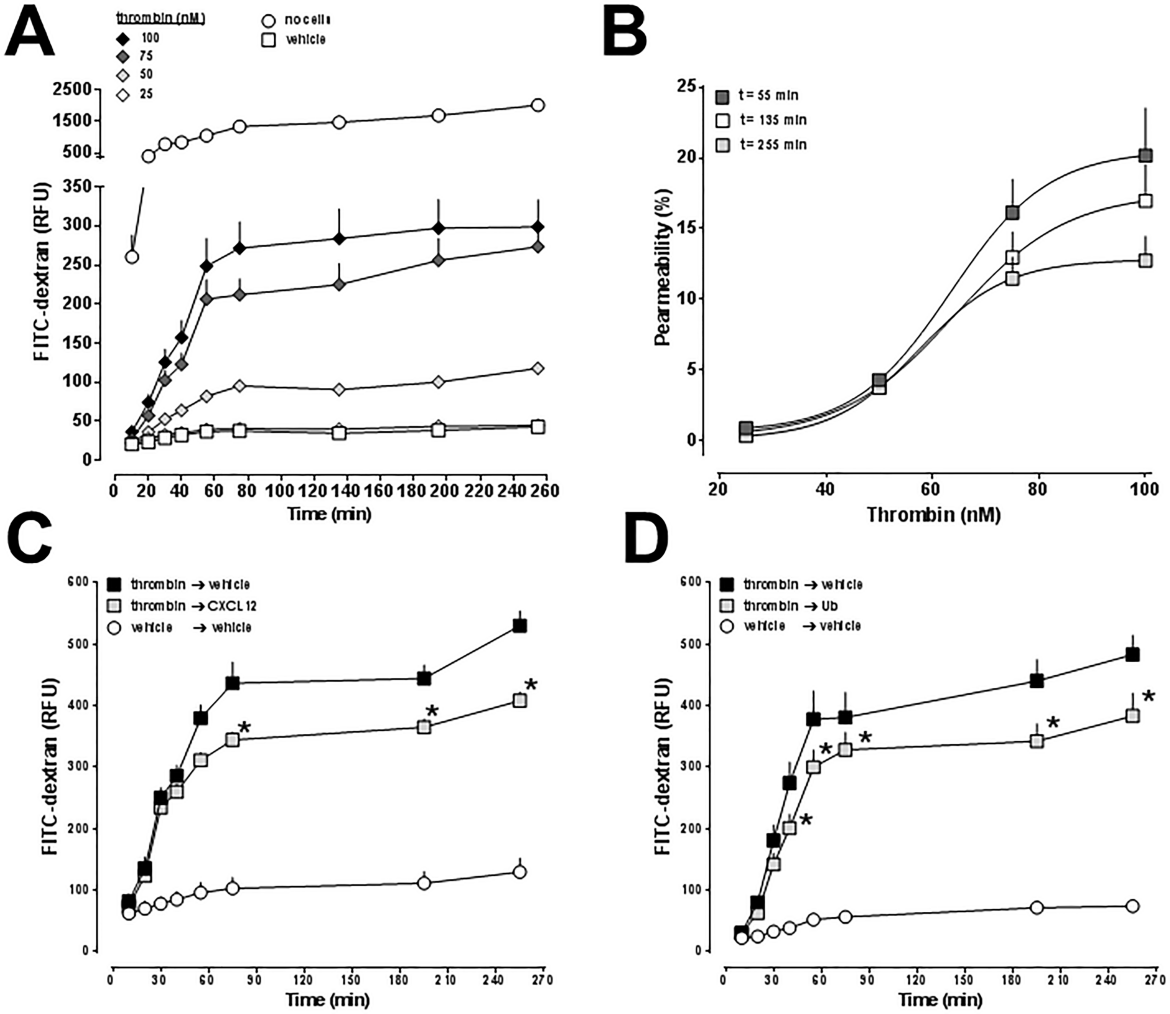
Effects of CXCL12 and ubiquitin on thrombin-induced impairment of HULEC-5a monolayer permeability. (A) HULEC-5a were grown to a confluent monolayer on collagen-coated permeable membranes and then exposed to different concentration of thrombin for 10 min, followed by the addition of FITC-dextran. Endothelial permeability was assessed by measuring the amount of FITC-dextran that permeated through the cell monolayer. No cells: 100% permeability. RFU: Relative fluorescence units. N = 3 in quadruplicate. (B) Dose-response curves for thrombin-induced permeability, data from A. 100% permeability = permeability in the absence of HULEC-5a. Open squares: Permeability at t = 55 min. Light grey squares: Permeability at t = 135 min. Dark grey squares: Permeability at t = 255 min. (C/D) HULEC-5a were grown to a confluent monolayer on collagen-coated permeable membranes and then exposed to 50 nM of thrombin or vehicle. After 10 min, thrombin-exposed cells were treated with vehicle, CXCL12 (50 nM) (C) or ubiquitin (50 nM) (D), followed by the addition of FITC-dextran. The experimental conditions are indicated. Endothelial permeability was assessed by measuring the amount of FITC-dextran that permeated through the cell monolayer. RFU: Relative fluorescence units. N = 3 in quadruplicate. *: p<0.05 vs. thrombin/vehicle (2-way ANOVA/Bonferroni’s multiple comparison post hoc test).

As observed in hPPAEC, thrombin also dose- and time-dependently induced permeability in the human lung microvascular endothelial cell line HULEC-5a (Fig 4A and 4B). When compared with hPPAEC, potency and efficacy of thrombin to induce permeability were reduced in HULEC-5a. The EC_50_ for thrombin-induced impairment of HULEC-5a barrier function was 64±7 nM after 55 min, 64±6 nM after 135 min and 57±5 nM after 255 min. Addition of CXCL12 and ubiquitin after treatment of HULEC-5a cells with a sub-maximal dose of thrombin significantly reduced thrombin-mediated impairment of endothelial cell barrier function (Fig 4C and 4D). The protective effects of both CXCR4 agonists on thrombin-induced permeability were comparable in HULEC-5a cells; CXCL12, however, was less efficacious in HULEC-5a cells than in hPPAEC.

**Fig 5:**
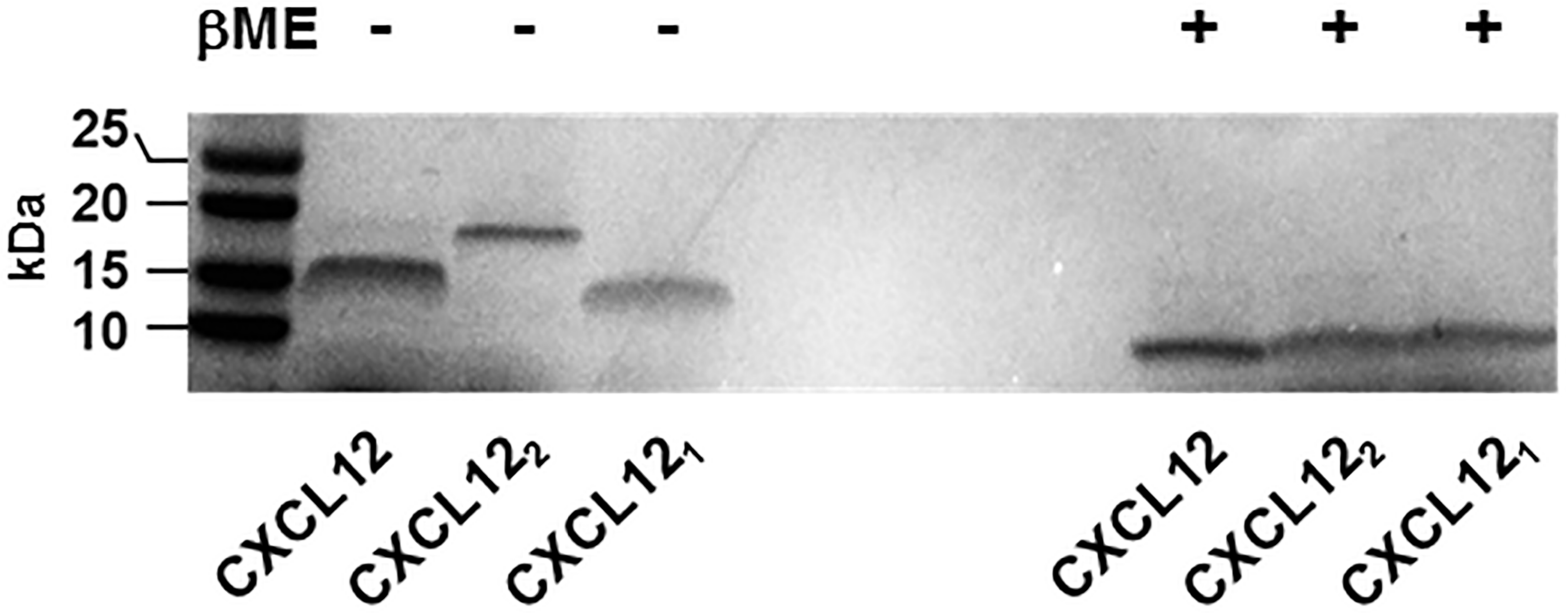
Electrophoretic mobility of CXCL12α, CXCL12_1_ and CXCL12_2_. Per lane 1 μg of protein in 25μL sample buffer (4 μM) were used for SDS-polyacrylamide gel electrophoresis under non-reducing (-) and reducing (+, βME: 0.357 M β-mercaptoethanol) conditions. The position of molecular mass standards is indicated on the left.

Next, we utilized the Presto-Tango β-arrestin 2 recruitment assay to assess CXCR4 and ACKR3 agonist activities of the natural splice variants CXCL12α and CXCL12β, of truncated CXCL12 (3–68) and the engineered constitutively monomeric (CXCL12_1_) and dimeric (CXCL12_2_) CXCL12 variants. To confirm the dimeric and monomeric structure of the CXCL12_1_ and CXCL12_2_ variants, we performed polyacrylamide gel electrophoresis (PAGE) under non-reducing and reducing conditions (Fig 5). Consistent with the mono- and dimeric nature of the CXCL12 variants [27, 29], the migration position of CXCL12_2_ was close to 20 kDa under non-reducing conditions, whereas CXCL12 and CXCL12_1_ migrated to a position corresponding to a lower molecular mass. It should be noted that a faint band migrating at the position of CXCL12_2_ was visible in non-reducing SDS-PAGE with CXCL12 (loaded at 4 μM), which is consistent with its dimerization *K*_d_ of 140 μM [42]. Under reducing conditions all three proteins showed an identical migration position, corresponding to the monomeric molecular mass of approximately 8 kDa.

**Fig 6:**
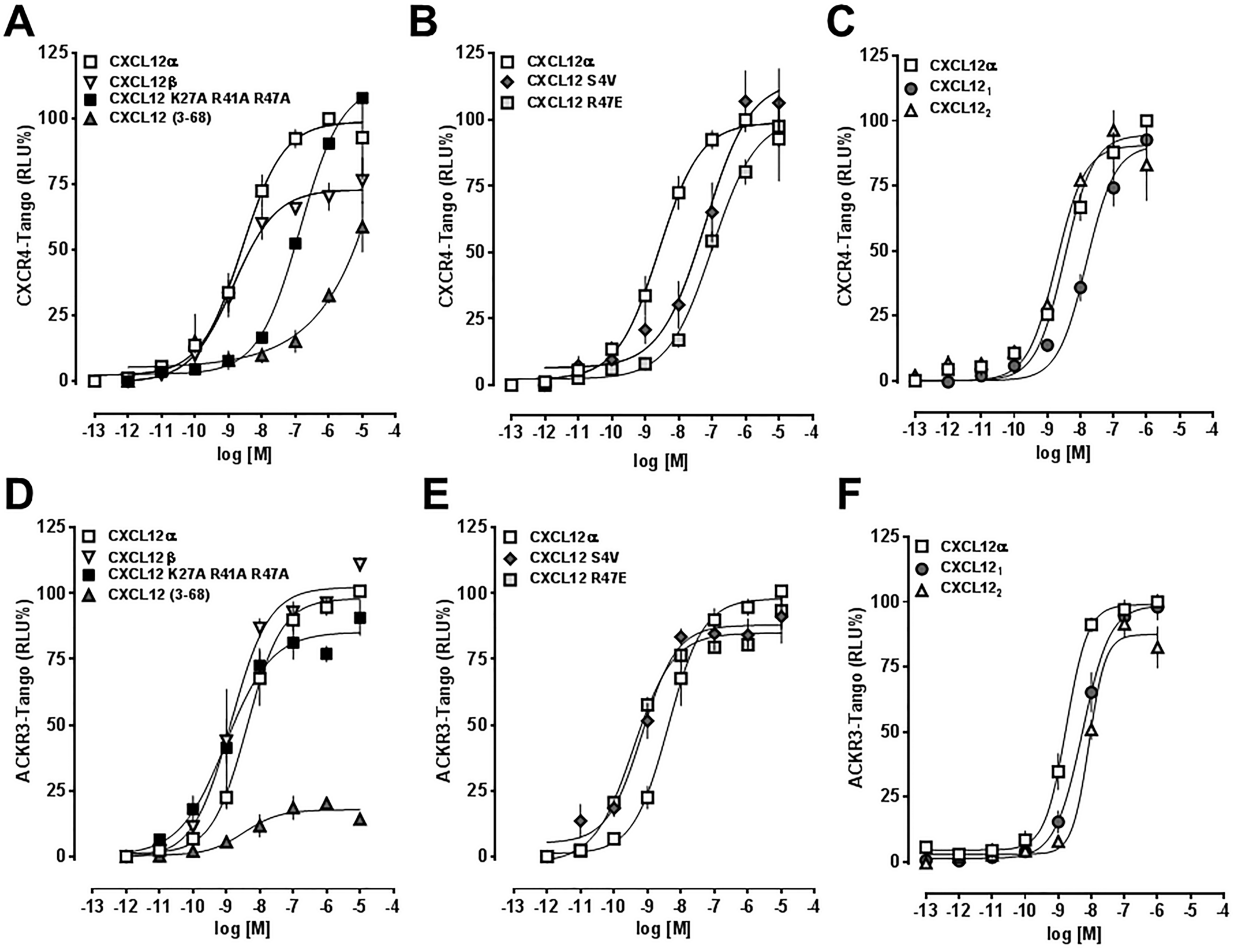
Presto-Tango β-arrestin 2 recruitment assays for CXCR4 (A-C) and ACKR3 (D-F). RLU%: % of the luminescence signal for 1 μM CXCL12α. N = 9 for CXCL12α and n = 3 for all other proteins.

In addition, we tested CXCL12 S-S4V, a protease resistant mutant, CXCL12 K27A/R41A/R47A, which shows significantly reduced heparan sulfate proteoglycan binding properties, and CXCL12 R47E, which activates CXCR4 with reduced potency, as compared with CXCL12α. The dose-response curves are shown in Fig 6 and Table 1 summarizes the corresponding EC_50_ concentrations and top plateau values (efficacy) for each protein.

**Table 1:** CXCR4 and ACKR3 activity of CXCL12/CXCL12 variants–PRESTO-Tango.

Protein	CXCR4		ACKR3	
	EC_50_ (nM)	efficacy %	EC_50_ (nM)	efficacy %
CXCL12α	5.18±4.4	100	5.8±8.6	100
CXCL12β	1.4±0.5	73±11	1.5±0.8	103±2
CXCL12_1_	27±11	103±10	5.6±2.1	98±8
CXCL12_2_	2.4±1.3	90±19	8.8±0.8	87±12
CXCL12 S-S4V	75±30 (0.047)	117±27	0.7±1.4	88±12
CXCL12 R47E	127±52 (<0.01)	105±18	0.4±0.02	86±7
CXCL12 K27A R41A R47A	145±95 (<0.01)	111±17	1.2±1.6	83±7
CXCL12 (3–68)	>10^3^	nd	13.3±13.5	19±2 (<0.01)

Data are mean±SD. %: Relative efficacy in % of the efficacy of CXCL12α (wild type). N = 9 for CXCL12α, n = 3 for all other proteins. Data were compared with 1-way ANOVA/Bonferroni post hoc testing. Statistically significant differences vs. CXCL12α are shown in parenthesis. nd: not determined.

All proteins except CXCL12_3-68_, which lacked relevant CXCR4 activity, showed comparable efficacy to recruit β-arrestin 2 to CXCR4. There were no statistically significant differences between the EC_50_ concentrations for CXCL12α, CXCL12β, CXCL12_1_ and CXCL12_2_ in the CXCR4 Presto-Tango assay. The potencies of CXCL12 S-S4V, CXCL12 R47E and CXCL12 K27A/R41A/R47A were significantly lower than the potency of CXCL12α to recruit β-arrestin 2 to CXCR4.

In contrast, all proteins induced β-arrestin 2 recruitment to ACKR3 with an EC_50_ in the low nM range (p>0.05 for all vs. CXCL12α). While the efficacy for β-arrestin 2 recruitment to ACKR3 was significantly reduced for CXCL12 (3–68), the efficacies of all other proteins for β-arrestin 2 recruitment to ACKR3 were comparable.

**Fig 7:**
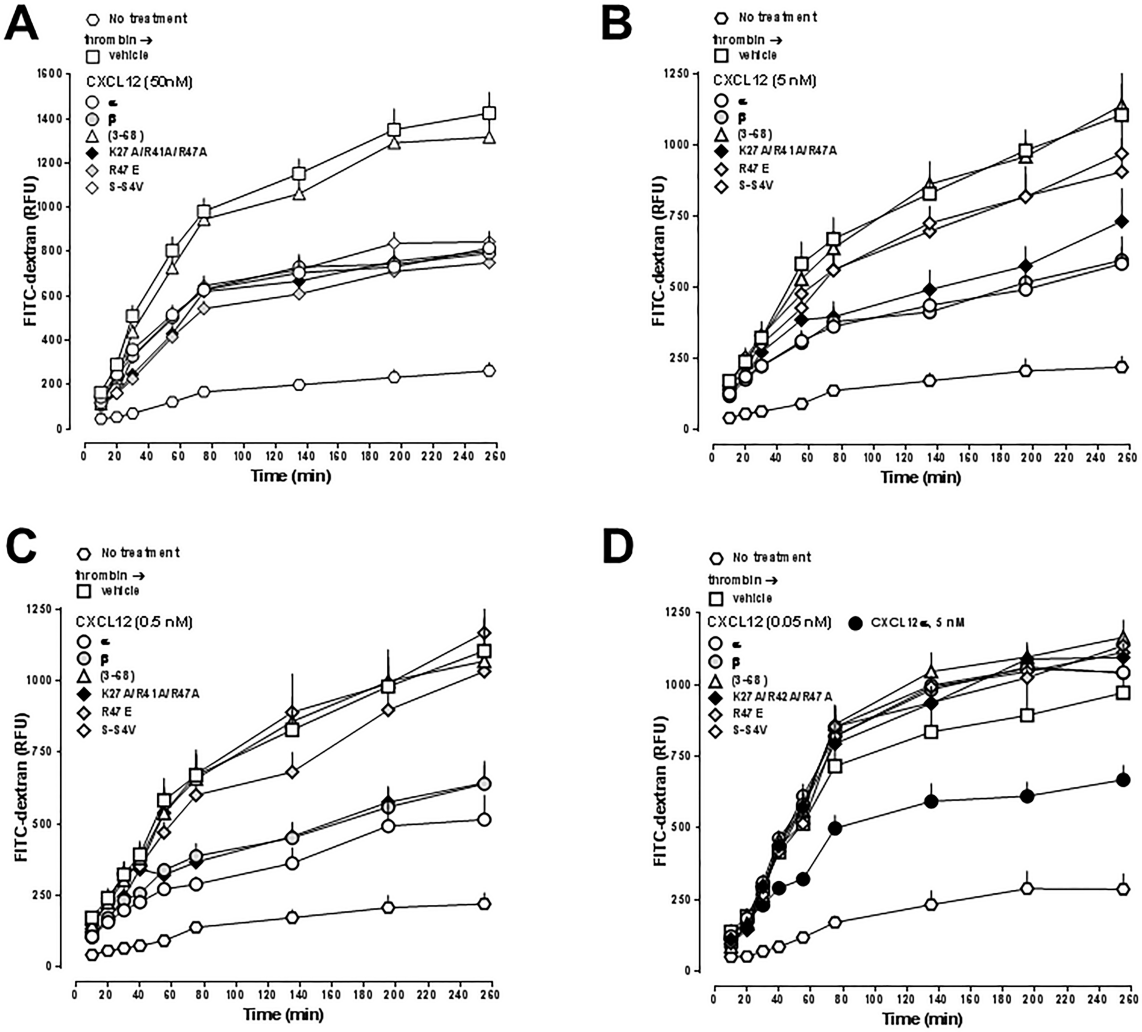
Dose-dependent effects of CXCL12α/β, CXCL12 (3–68) and CXCL12 mutants K27A/R41A/R47A, R47E and S-S4V on thrombin-induced impairment of hPPAEC monolayer permeability. hPPAEC cells were grown to a confluent monolayer on collagen-coated permeable membranes. hPPAEC were then exposed to 35 nM of thrombin. After 10 min, thrombin-exposed cells were treated with vehicle or 50 nM (A), 5 nM (B), 0.5 nM (C) or 0.05 nM (D) of the various proteins, as indicated. In (D) 5 nM CXCL12α was used as a positive control. N = 3 in quadruplicate.

**Fig 8:**
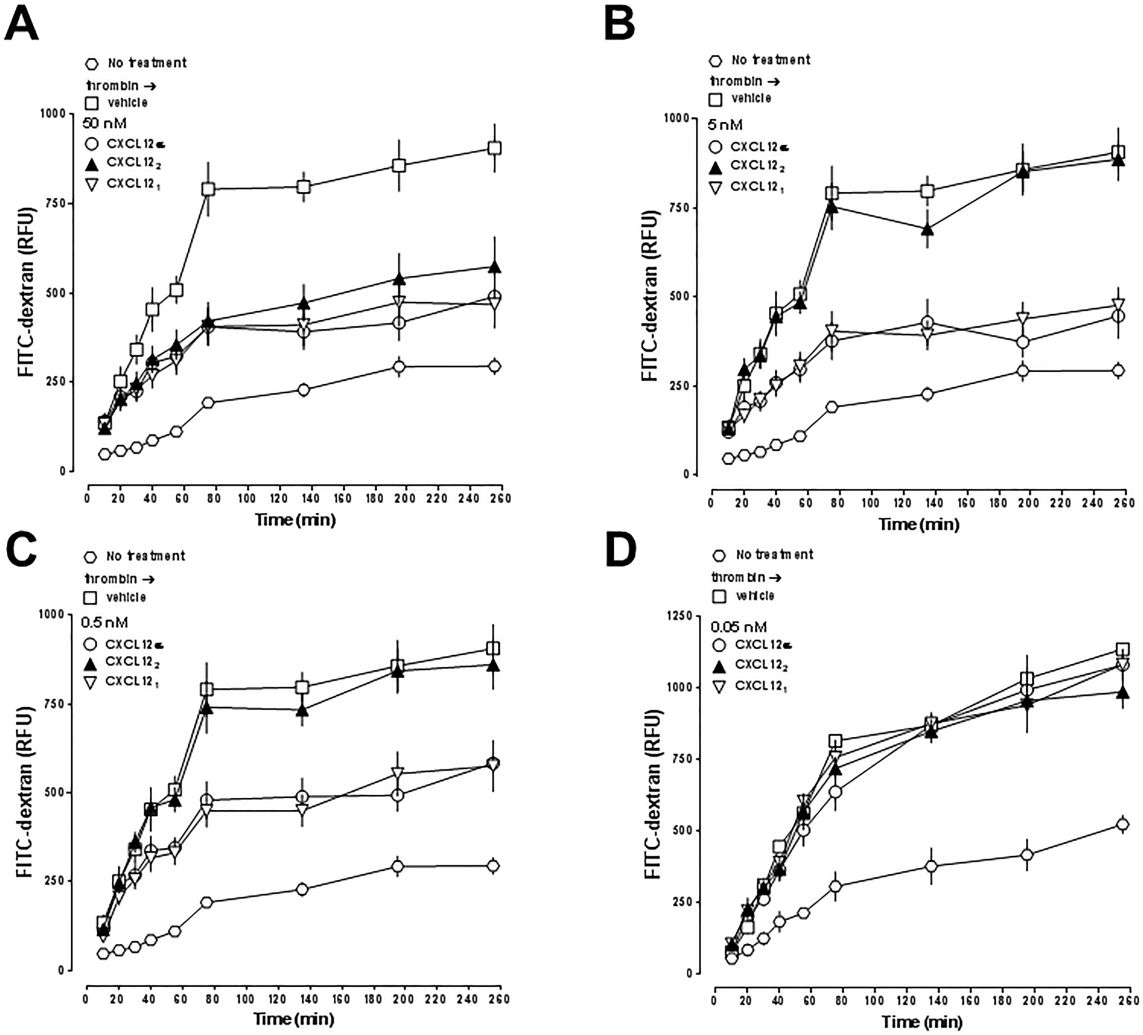
Dose-dependent effects of CXCL12α, CXCL12_1_ and CXCL12_2_ on thrombin-induced impairment of hPPAEC monolayer permeability. hPPAEC cells were grown to a confluent monolayer on collagen-coated permeable membranes. hPPAEC were then exposed to 35 nM of thrombin. After 10 min, thrombin-exposed cells were treated with vehicle or 50 nM (A), 5 nM (B), 0.5 nM (C) or 0.05 nM (D) of the various proteins, as indicated. In (D) 5 nM CXCL12α was used as a positive control. N = 3 in quadruplicate.

Figs 7 and 8 show the effects of the proteins on thrombin-mediated impairment of hPPAEC barrier function when tested in concentrations between 0.05–50 nM in parallel experiments. CXCL12 (3–68) did not attenuate thrombin-induced impairment of hPPAEC barrier function. All other proteins inhibited thrombin-mediated impairment of hPPEAC barrier function with a similar time-dependency of their effects.

**Fig 9:**
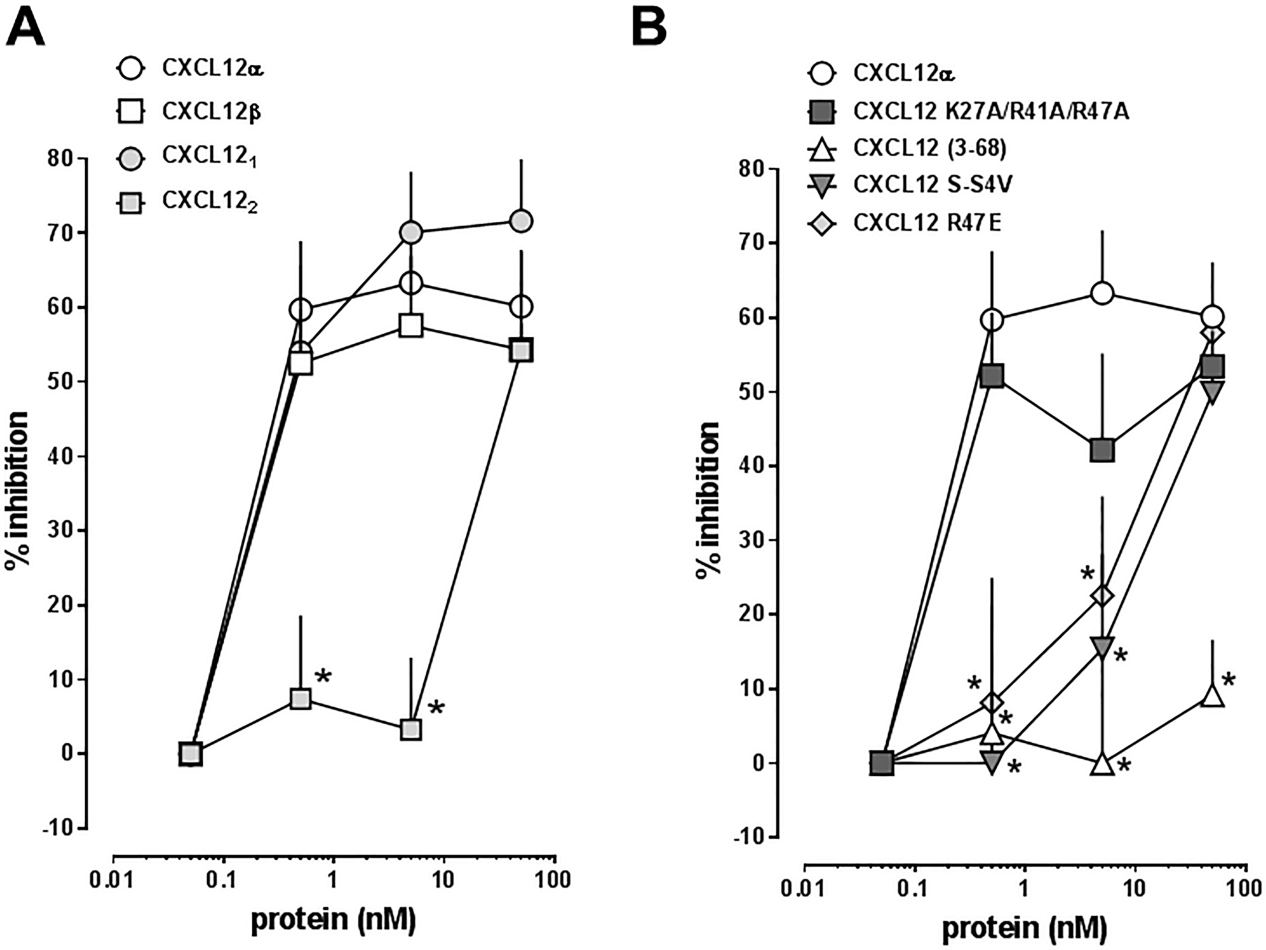
Inhibition of thrombin-induced hyper-permeability of hPPAEC by CXCL12/CXCL12 variants. % inhibiton: % inhibiton of thrombin-induced hyperpermeability. Data from Figs 7 and 8 at t = 255 min. N = 3 inquadruplicate. *: p<0.05 vs. CXCL12α (2-way ANOVA/Bonferroni’s multiple comparison post hoc test).

Fig 9 shows the comparison of their end-point (t = 255 min) dose-response profiles. Except for CXCL12 (3–68), the efficacies of all other proteins to inhibit thrombin-induced impairment of hPPAEC barrier function were comparable. CXCL12α, CXCL12β, CXCL12_1_ and CXCL12 K27A/R41A/R47A showed similar potencies to inhibit thrombin-mediated hyper-permeability of hPPAEC with EC_50_ concentrations between 0.05–0.5 nM. The potencies of CXCL12 R47E and CXCL12 S-S4V were one order of magnitude lower (EC_50_ between 0.5–50 nM). CXCL12_2_ affected thrombin-mediated hPPAEC barrier impairment only at a concentration of 50 nM.

## Discussion

In the present study, we evaluated the effects of CXCR4 and ACKR3 ligands on the barrier function of human lung endothelial cells. CXCL12 has previously been described to enhance transendothelial electrical resistance, a surrogate marker of endothelial barrier function, of bovine aortic, human pulmonary artery and umbilical vein endothelial cells [22]. Furthermore, pre-treatment of bovine aortic endothelial cells with CXCL12 has been reported to attenuate thrombin-induced FITC-dextran transfer in transwell permeability assays. Likewise, co-treatment of human microvascular endothelial cells with CXCL12 or CTCE-0214, a synthetic CXCL12 analogue, plus thrombin attenuated the reduction of transendothelial resistance that was detectable with thrombin alone [15, 22]. Our observations from the present study are in agreement with previous reports and now provide direct evidence that CXCL12 enhances barrier function of hPPAEC in the absence of permeability-inducing agents. In addition, we demonstrate that pre-treatment of hPPAEC with CXCL12 and with the non-cognate CXCR4 agonist ubiquitin, which does not bind to ACKR3 [38], attenuates thrombin-mediated hPPAEC barrier function impairment. These findings provide a possible mechanism underlying lung protective effects of intravenous CXCL12 pre-treatment in an oleate-induced lung injury model in rabbits and of ubiquitin pre-treatment in an endotoxic shock model in pigs [17, 20].

Although pre- and co-treatment experiments provide information on possible preventive properties of CXCR4 agonists, such experiments are unable to address therapeutic potential. Thus, we performed post-treatment experiments and detected that activation of CXCR4 after thrombin-exposure of hPPAEC and HULEC5a cells attenuates thrombin-mediated impairment of lung endothelial barrier function. These findings support the concept that CXCR4 agonists have therapeutic potential to limit thrombin-mediated pulmonary vascular leakage, which likely contributed to lung protective effects of CXCR4 agonists that have been observed in various models when administered after the insult [15, 17, 18, 21, 43].

In contrast to CXCL12, the non-cognate CXCR4 agonist ubiquitin did not enhance hPPAEC barrier function in the absence of thrombin. As compared with CXCL12, ubiquitin was less efficacious to reduce thrombin-mediated barrier function impairment in pre-treatment experiments with hPPAEC, showed similar efficacy to protect barrier function after thrombin exposure of HUELC5a and failed to protect barrier function after thrombin exposure of hPPAEC. These findings could be explained by ubiquitin’s lower affinity for and weaker agonist activity at CXCR4, as compared with CXCL12 [38, 39, 44–46].

Recently, we provided evidence that ubiquitin functions as a biased CXCR4 agonist, which does not recruit β-arrestin 2 to CXCR4 [47]. Thus, it appears also possible that the differences between CXCL12 and ubiquitin that we observed in the present study reflect differences in functional outcomes of balanced and biased CXCR4 signaling in lung endothelial cells.

Because none of the ACKR3 agonists affected hPPAEC barrier function and AMD3100 abolished protective effects of CXCL12 on thrombin-mediated barrier function impairment, activation of ACKR3 alone appears not to contribute to the observed effects.

Previously, CXCR4 has been shown to form heteromeric complexes with ACKR3 in expression systems and in human vascular smooth muscle cells [34–36, 48, 49]. Our present finding that PLA signals for CXCR4 and ACKR3 interactions are also detectable in hPPAEC suggests the existence of such endogenous receptor heteromers in the lung endothelium. Thus, another explanation for the observed differences between CXCL12 and ubiquitin could be that simultaneous activation of CXCR4 and ACKR3 within the heteromeric complex is more efficacious to reduce thrombin-mediated endothelial barrier impairment than activation of CXCR4 alone. To address this possibility, detailed mechanistic studies to elucidate the roles of the CXCR4:ACKR3 heteromer will be required in the future. Such experiments, however, are beyond the scope of the present study.

Among the CXCL12 variants that we tested, only CXCL12 (3–68) lacked relevant CXCR4 activity, showed significantly reduced efficacy to activate ACKR3 in Presto-Tango assays and did not attenuate thrombin-induced hPPAEC barrier function impairment. This loss of function is consistent with the loss of function of N-terminal truncated CXCL12 that has been reported previously in other assay systems [23–25].

As expected, both natural CXCL12 splice variants, CXCL12α and CXCL12α, showed comparable properties in Presto-Tango and permeability assays [26]. CXCL12 exists as a monomer at low concentrations and forms dimers at high concentrations or when bound to heparan sulfate on the endothelial surface [31, 50, 51]. Consistent with previous reports, the constitutive monomeric CXCL12 variant (CXCL12_1_) showed a behavior similar to wild type proteins in CXCR4/ACKR3 β-arrestin 2 recruitment assays and in permeability assays [27, 28].

Despite activities of the disulfide-locked dimeric CXCL12 variant CXCL12_2_ in β-arrestin 2 recruitment assays for CXCR4 and ACKR3 that were comparable with CXCL12α/β, CXCL12_2_ showed significantly reduced potency to attenuate thrombin-induced permeability of hPPEAC. The previous finding that CXCL12_2_ binds to ACKR3 with very low affinity is not contradictive to our findings in ACKR3 β-arrestin 2 recruitment assays because maximal biological responses of other GPCRs have been observed at ligand occupancies of only a small fraction of receptors [28, 52, 53] and a large receptor reserve is likely in expression systems, such as the Presto-Tango assay. The effects of CXC12_2_ in CXCR4 β-arrestin 2 recruitment assays that we observed using the Presto-Tango cell system, however, are conflicting with previous measurements in intermolecular bioluminescence resonance energy transfer (BRET) assays [28]. The Presto-Tango assay utilizes a transcriptional read-out that is measured several hours after the actual signaling event. Thus, it appears possible that few β-arrestin recruitment events upon ligand binding, which may not generate a significant intermolecular BRET signal, can lead to transcription of luciferase in the Presto-Tango system. Furthermore, as compared to previous intermolecular BRET assays in which cells were exposed to CXCL12_2_ for 30 min [28], cells were exposed to CXCL12_2_ in our Presto-Tango assays for longer time periods, which may contribute to the observed effects. Irrespective of this discrepancy, the low potency of CXCL12_2_ to inhibit thrombin-mediated barrier function impairment in the present study in combination with the previously described lack of chemotactic activity of CXCL12_2_ [28, 29] demonstrate that this variant does not induce the complete spectrum of biological effects that are mediated via CXCR4 and/or ACKR3 upon activation with the wild type proteins and the constitutively monomeric variant.

In agreement with the low potency of CXCL12 R47E to activate Ca^2+^ signaling via CXCR4 [29], we observed that this mutant also induces β-arrestin 2 recruitment to CXCR4 and antagonizes thrombin-mediated hyperpermeability of hPPAEC with reduced potency, but retains ACKR3 activity comparable to wild type proteins. Similarly, the protease resistant mutant CXCL12 S-S4V showed reduced CXCR4 activity in Presto-Tango and permeability assays but retained ACKR3 activity. These findings are in agreement with previous effects of this mutant in CXCR4/ACKR3 β-arrestin recruitment and chemotaxis assays [30]. The observations that CXCL12 R47E and CXCL12 S-S4V showed reduced CXCR4 activity but retained ACKR3 activity further supports the assumption that protection from thrombin-mediated hPPAEC barrier impairment is mediated via CXCR4.

CXCL12 is known to bind to heparin oligosaccharides, which promotes dimerization, interferes with CXCL12 binding to CXCR4 and immobilizes CXCL12 on the endothelial surface to establish a concentration gradient required for cell trafficking [31, 54–56]. CXCL12 K27A/R41A/R47A, which binds heparan sulfates with significantly reduced affinity [31], was the only mutant protein that inhibited thrombin-mediated impairment of hPPAEC barrier function with that same potency as wild type proteins and CXCL12_1_. This mutant, however, showed the lowest potency to activate CXCR4 in β-arrestin 2 recruitment assays and retained high potency to activate ACKR3. These data suggest that the K27, R41 and R47 mutations reduced the binding affinity for CXCR4 or the efficacy to induce signaling events at lower concentrations. The high potency of this mutant to reduce thrombin-induced barrier function impairment, however, can be explained by its reduced heparan sulfate binding properties, which reduces the proportion of protein that is immobilized on the surface of hPPAEC and thus, is not available for receptor activation [57]. The latter suggests that CXCL12 binding to heparan sulfate on HTLA cells, which were used in Presto-Tango assays, does not significantly affect CXCR4 binding and signaling. This implies that distinct cell surface heparan sulfate proteoglycan expression patterns between various cell types modulate CXCL12-mediated biological functions.

In conclusion, our findings suggest CXCR4 as a possible drug target to attenuate thrombin-mediated impairment of lung endothelial barrier function, demonstrate that stimulation of human lung endothelial cells with cognate and non-cognate CXCR4 agonists results in functional differences and provide initial information on the structure-function relationship for CXCL12-mediated protection from thrombin-induced barrier function impairment in primary human lung endothelial cells. Our findings indicate that the protective effects of CXCL12 are dictated by its CXCR4 agonist activity and by interactions of distinct protein moieties with heparan sulfate proteoglycans on the endothelial cell surface. Interestingly, in disease conditions that are likely associated with thrombin-induced endothelial permeability impairment, such as sepsis or trauma, systemic CXCL12 concentrations have been reported to increase to levels within the range of the EC_50_ for CXCL12 to attenuate thrombin-induced barrier function impairment in our permeability assays [58–60]. This may suggest that activation of CXCR4 by its endogenous agonists constitutes a protective mechanism to attenuate endothelial barrier function impairment by thrombin in disease conditions and implies that treatment with exogenous CXCR4 agonists augments this protective response. Our findings are expected to facilitate the development of engineered compounds with improved pharmacological properties to attenuate thrombin-induced vascular leakage in the pulmonary circulation, which may have the potential to attenuate development of lung injury and ARDS.

## Supporting information

S1 Dataset All data sets are provided in this file “S1 data sets.xlsx”. (XLSX)
